# Transcriptome analysis reveals dysregulation of innate immune response genes and neuronal activity-dependent genes in autism

**DOI:** 10.1038/ncomms6748

**Published:** 2014-12-10

**Authors:** Simone Gupta, Shannon E. Ellis, Foram N. Ashar, Anna Moes, Joel S. Bader, Jianan Zhan, Andrew B. West, Dan E. Arking

**Affiliations:** 1 Department of Medicine, McKusick-Nathans Institute of Genetic Medicine, Johns Hopkins University School of Medicine, Baltimore, Maryland 21205, USA; 2 Department of Biomedical Engineering, Johns Hopkins University School of Medicine, Baltimore, Maryland 21205, USA; 3 Department of Neurology, University of Alabama at Birmingham, Birmingham, Alabama 35294, USA

## Abstract

Recent studies of genomic variation associated with autism have suggested the existence of extreme heterogeneity. Large-scale transcriptomics should complement these results to identify core molecular pathways underlying autism. Here we report results from a large-scale RNA sequencing effort, utilizing region-matched autism and control brains to identify neuronal and microglial genes robustly dysregulated in autism cortical brain. Remarkably, we note that a gene expression module corresponding to M2-activation states in microglia is negatively correlated with a differentially expressed neuronal module, implicating dysregulated microglial responses in concert with altered neuronal activity-dependent genes in autism brains. These observations provide pathways and candidate genes that highlight the interplay between innate immunity and neuronal activity in the aetiology of autism.

## Abstract (web summary)

Autism spectrum disorder (ASD) is a common, highly heritable neurodevelopmental condition characterized by marked genetic heterogeneity. In this study, the authors use RNA sequencing analyses to characterize differences in the transcriptome between autistic and typically developing brains.

## Graphical abstract

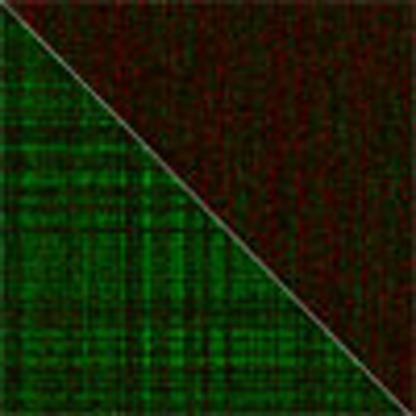

Autism is a neurodevelopmental disorder in which affected individuals show disruption of the normal course of social and communicative development, along with restrictive interests and repetitive behaviours. In the vast majority of cases, the cause of disease is not known. Despite a strong genetic component with heritability estimates between 50 and 90% (refs 1, 2, 3), the identification of genes important in disease susceptibility has progressed slowly, resulting in a limited understanding of the molecular basis of autism. Environmental factors underlying disease risk are likewise unclear. This slow progress can be attributed, in part, to both the extreme phenotypic heterogeneity and involvement of hundreds of loci in the disorder^4^,^5^.

Recent studies to elucidate the molecular basis of autism have largely focused on genetic approaches, including both genome-wide association studies^6^ and whole-exome sequencing^5^,^7^,^8^,^9^, to identify both inherited and *de novo* variation contributing to autism. A major mode of action for genetic variation is through altered gene expression, so direct analysis of gene expression in a disease-relevant tissue is a complementary approach to genetic studies. Despite the extreme genetic heterogeneity observed in autism, it is possible that common downstream mechanisms may be altered^10^. Thus, there has been an effort to use transcriptomics to identify and dissect molecular pathways that may be altered in autism spectrum disorder (ASD).

Given low tissue sample availability in autism research, efforts have focused on assessing gene expression in lymphoblastoid cell lines or whole blood^11^,^12^,^13^,^14^,^15^. However, given the core neurodevelopment phenotypes associated with autism, there is little doubt that direct assessment of gene expression in brains may be critical. Indeed, Voineagu *et al*. recently utilized co-expressed gene networks from RNA-Sequencing (RNA-Seq) carried out in 19 autism brains and 17 controls to identify a set of co-expressed neuronal genes enriched for known autism susceptibility genes as well as a set of co-expressed genes enriched for both immune genes and glial markers^10^.

In the current study, we present results from the largest RNA sequencing of autism brains effort to date that allows for new insights into the aetiology of autism. We find clear differences in the transcriptome between control and ASD cortical brains. Using co-expression network analysis, we demonstrate that autism brains are specifically enriched for ‘activated’ M2 microglial and ‘immune response’ genes. Remarkably, the M2 microglial module is strongly negatively correlated with one of two differentially expressed neuronal modules, highlighting the interplay between innate immunity and neuronal activity in the aetiology of ASD.

## Results

**Figure 1:**
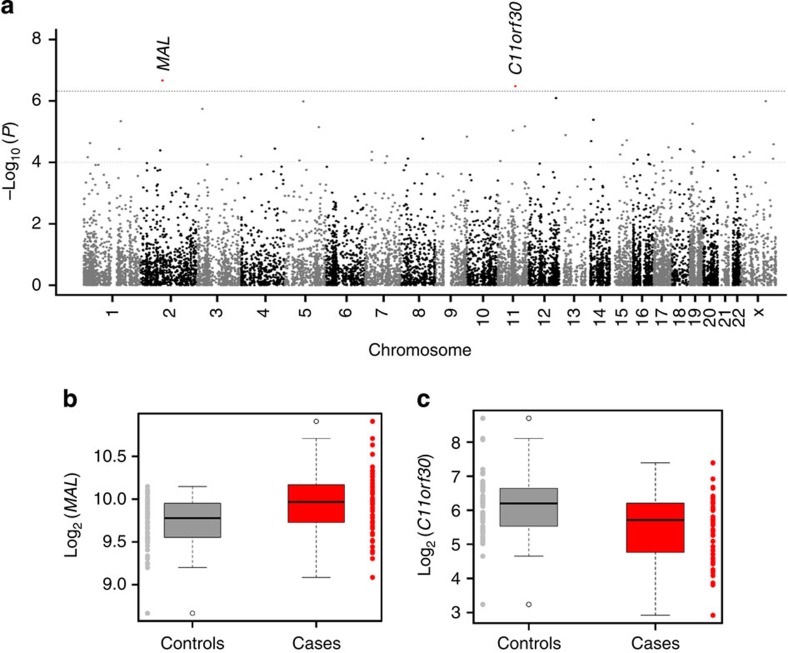
Single-gene expression analysis identifies two transcriptome-wide significantly differentially expressed genes between autism and control brains. (**a**) Manhattan plot for 13,262 expressed genes. The threshold for transcriptome-wide significance was calculated based on 400 permutations (*P*<4.76 × 10^−7^) and is indicated by the dotted grey line. (**b**,**c**) Boxplot of gene expression in 57 controls (grey) and 47 cases (red), indicating a 1.2-fold increase for *MAL* and a 0.6-fold decrease for *C11orf30* in cases relative to controls.

Transcriptomes from 104 human brain cortical tissue samples were resolved using next-generation RNA sequencing technology at single-gene resolution and through co-expressing gene clusters or modules. Multiple cortical tissues corresponding to Brodmann Area 19 (BA19), Brodmann Area 10 (BA10) and Brodmann Area 44 (BA44) were sequenced in 62, 14 and 28 samples, respectively, resulting in a total of 57 (40 unique individuals) control and 47 (32 unique individuals) autism samples (Supplementary Data 1 and 2, and see ref. 16). Differential gene expression was estimated between the 57 controls and the 47 cases, with sample collection site, age, sex, brain region and independent surrogate variables (ISVs) as fixed effects in a linear mixed regression model (Supplementary Data 3a). In total, 13,262 genes with at least three reads per sample across 90% of the samples were tested, and two transcriptome-wide significant differentially expressed genes associated with autism were identified (Supplementary Data 4 and Fig. 1a).

The most significant differentially expressed gene was *Myelin And Lymphocyte Protein* (*MAL*; *P*=2.16 × 10^−7^, Fig. 1b, and Supplementary Fig. 1a–c). *MAL*, along with other myelination genes, has previously been reported to show altered expression in patients with psychiatric disorders^17^. The second differentially expressed gene was *C11orf30* (*EMSY*; *P*=3.29 × 10^−7^, Fig. 1c and Supplementary Fig. 1d–f). This gene has been implicated in chromatin modification, DNA repair and transcriptional regulation, and previous genome-wide association studies have linked *C11orf30* to inflammatory and malignant diseases^18^,^19^. We also performed analyses at the exon level, testing 21,310 exons for differential exon expression; however, no differences could be identified in autism cases and controls after correction for multiple testing (Supplementary Data 5).

Next we asked whether the top differentially expressed genes from the single-gene analysis shared common pathways or functional categories. We tested for the enrichment of biological processes using Gene Ontology (GO) annotations and MSigDB curated gene sets. No gene-set enrichment with family-wise error rate (FWER) ≤0.05 was observed (Supplementary Data 6a–d).

For common genetic variation, altered gene expression is a major mode of action^20^,^21^. We therefore tested whether genes previously associated with autism through genetic analyses are enriched for altered gene expression. To identify genes underlying susceptibility to autism, we utilized a list of expertly curated genes developed by the Simons Foundation for Autism Research (SFARI)^22^. In addition to the SFARI list of genes, we also integrated genes associated with rare *de novo* variation (RDNV) and genes involved in intellectual disability (ID) compiled from four published whole-exome sequencing studies^5^,^7^,^8^,^23^ and review articles^24^ (Supplementary Data 7). Overall, we find comparable expression of these genes in autism and control brain tissue (Supplementary Data 8a,b). Although these gene lists are not comprehensive and only reflect the current understanding of the genetic basis of autism, the lack of enrichment for genes known to harbour genetic signal for autism in altered gene expression suggests the potential for non-overlapping mechanisms between genetic and transcriptomic determinants of autism. However, we do find modest enrichment for a broader set of genes containing ‘brain-critical exons’, which have high gene expression in brain and low rare burden of rare mutations, and have been proposed to represent autism candidate genes^25^ (Supplementary Data 8a).

**Figure 2:**
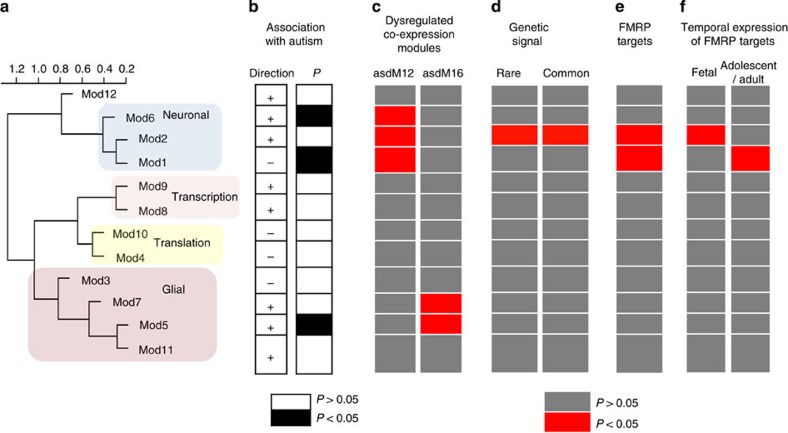
Weighted gene correlation network analysis identifies 12 co-expression modules. (**a**) Dendogram of 12 co-expressed modules, with major cell type/function enrichment noted (Supplementary Data 12,13a–k). Mod12 was not significantly enriched for any cell type or GO terms. (**b**) Disease association of each module, represented by each module’s first principal component (eigengene). Three co-expression modules are associated with autism with nominal significance (black), with mod5 significant after multi-test correction (*P*<9.64 × 10^−4^; Supplementary Data 11). A positive (+) sign indicates upregulated gene expression in autism cases. (**c**–**f**) Enrichment analysis for gene lists compiled from the literature (Supplementary Data 7,12). (**c**) Individual dysregulated co-expression modules in autism brains identified by Voineagu *et al*.^10^ are captured by multiple co-expression modules in the current study, allowing for refinement of the signal associated with autism. (**d**) Genes with known common and rare *de novo* variants associated with autism are enriched only for mod2, which does not show differential expression. (**e**) FMRP targets identified by Darnell *et al*.^35^ are enriched in neuronal co-expression modules. (**f**) FMRP targets were split into fetal and adult/adolescent expression patterns^36^, and are captured by different co-expression modules. Red, *P*<0.05; grey, *P*>0.05.

**Figure 3:**
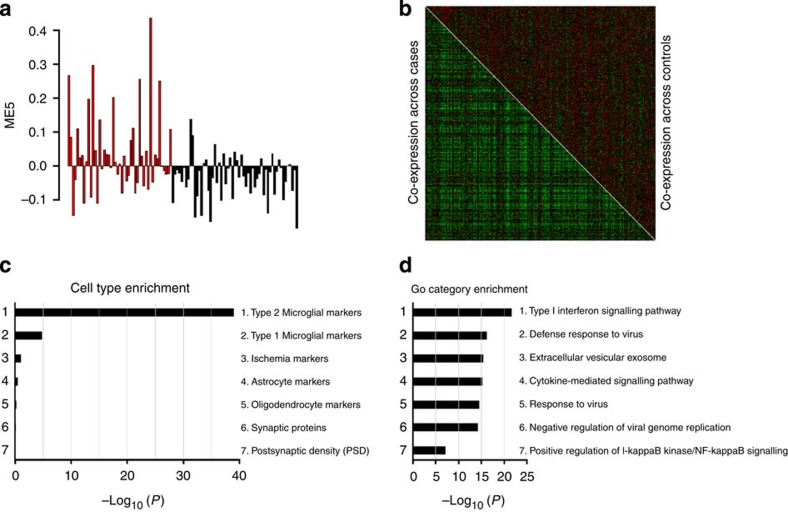
Gene co-expression module mod5 is associated with autism. (**a**) The module eigengene of mod5 is upregulated in autism cases (red) compared with controls (black). (**b**) Heatmap of mod5 co-expression for 759 genes, stratified by disease status, showing greater co-expression between cases (bottom left) compared with controls (upper right). (**c**) Mod5 is significantly enriched for genes associated with M2-microglial cell states (Supplementary Data 12). (**d**) Mod5 is significantly enriched for GO terms related to immune response (Supplementary Data 13e).

In addition to the single-gene analyses, we applied weighted gene correlation network analysis^18^ (WGCNA) to identify discrete gene modules based on co-expression between genes. Considerable overlap was observed in the modules constructed separately from cases and controls, indicating that overall organization of transcript co-expression is conserved between autism and control brains (Supplementary Data 9). Therefore, we applied WGCNA to construct networks derived from the entire data set of 104 samples, adjusted for sequencing artefacts, age, sex, collection site and brain region, identifying 12 co-expressed modules (Supplementary Data 10 and Fig. 2a). We tested the association of each module, represented by its corresponding first principal component or module eigengene (ME), with case–control status using a linear mixed regression framework (Supplementary Data 11 and Fig. 2b). Three of the twelve modules were differentially co-expressed (*P*<0.005), with mod5 (*P*=9.64 × 10^−4^) exceeding the multitest correction threshold (*P*_permutated_<0.002, *P*_Bonferroni_<0.004; Fig. 3a,b). Mod5 comprised 759 genes (Supplementary Fig. 2a) with enrichment for M2-microglial cell states (*P*_hypergeometric_=1.22 × 10^−39^; Supplementary Data 12 and Fig. 3c) and the GO term ‘Type I Interferon pathway’ (*P*_hypergeometric_=1.19 × 10^−20^; Supplementary Data 13e and Fig. 3d). Type I Interferon responses in the brain are classically attributed to viral infections that can produce M1 activation states in microglia^26^. Accordingly, mod5 also shows enrichment for GO terms ‘defense response to virus’ (*P*_hypergeometric_=6.83 × 10^−17^) and ‘cytokine-mediated signalling pathway’ (*P*_hypergeometric_=6.31 × 10^−16^; Supplementary Data 13e). In opposition to M1-activated microglia, M2 responses are responsible for mediating anti-inflammatory remediation responses to damage caused by viral infections. M2 microglial cells also secrete brain-derived neurotrophic factor (BDNF), increase the production of neural progenitor cells and promote myelination^27^,^28^,^29^. These data provide support for a mechanistic connection for viral-infection hypotheses^30^ for autism with neural over-growth hypotheses^31^ through the novel identification of exaggerated M2 activation states in autism brain tissue.

Voineagu *et al*. previously reported a co-expression module dysregulated in autism brains, termed asdM16, enriched in astrocytes and microglia-expressed genes^10^. To better understand the functional implications of asdM16 in autism, we looked for asdM16 signal enrichment among the modules generated utilizing our substantially larger data set (Supplementary Data 12 and Fig. 2c). Two modules—mod5 (*P*_hypergeometric_=9.3 × 10^−59^, described above) and mod7 (*P*_hypergeometric_=1.45 × 10^−89^)—were enriched for asdM16 signal. However, mod7 is not differentially expressed with respect to autism (Supplementary Data 11 and Fig. 2b) and accounts for the astrocyte markers (*P*_hypergeometric_=1.65 × 10^−75^; Supplementary Data 12), whereas mod5 is differentially expressed (*P*=9.64 × 10^−4^). By substantially increasing the sample size and number of genes evaluated, we are able to accurately pinpoint the relevant signal from the previously reported asdM16 module as coming from M2-state microglial cells and immunogenic responses (type I interferon responses; Supplementary Data 13e and Fig. 3d), and not from astrocytes. To our knowledge, M2 activation state responses have not previously been attributed to the pathogenesis of autism.

We also identified three distinct modules (mod1, mod2 and mod6; Supplementary Fig. 3b–d) enriched for neuronal markers that contain genes with the shared GO term, ‘synaptic transmission’, all of which showed enrichment for an additional co-expression module reported to be dysregulated in autism, asdM12 (Supplementary Data 12 and 13a,b,f, and Fig. 2c). Two of the three modules—mod1, downregulated in autism, and mod6, upregulated in autism—were nominally differentially co-expressed between the autism and control brain samples (*P*<0.005; Supplementary Data 11 and Fig. 2b). Mod1 contains synaptic transmission genes enriched in GABA-related ion channel activity, whereas mod6 contains genes enriched in peptide and hormone signalling (Supplementary Fig. 3).

Previous studies have identified an enrichment of genetic association signals in genes selectively expressed in neurons^10^,^25^,^32^,^33^. Here we find that mod2 was enriched for both common (*P*_hypergeometric_=2.49 × 10^−6^) and rare classes of autism genetic variants (*P*_hypergeometric_=4.29 × 10^−4^) but comparably expressed between cases and controls (Supplementary Data 11 and 12, and Fig. 2d). That neuronal genes genetically associated with autism do not appear to have altered expression (mod2), coupled with the observation that neuronal genes without genetic signal do appear to be differentially expressed (mod1 and mod6), suggests that autism-associated differentially expressed genes are separable from genetic determinants of autism. Corroborating this idea, a recent study of gene networks in coronary artery disease has shown that genes at the centre of the networks, referred to as ‘key drivers’, were largely not genome-wide association study signal genes, suggesting that key regulatory genes may not harbour common inherited variation because of natural selection^34^.

Direct evidence for the role of Fragile X mental retardation protein (FMRP) in autism has been provided by Darnell *et al*. These authors reported that many of the protein-interacting partners of FMRP harbour ASD-associated common variants^35^. Similarly, Iossifov and colleagues reported an enrichment of ASD RDNVs in FMRP targets^5^. We therefore investigated whether FMRP targets were enriched in any of the co-expression modules detected in autism brain tissue. We report a 20% enrichment of FMRP targets in one of the differentially co-expressed neuronal modules, mod1 (*P*_hypergeometric_=1.80 × 10^−10^), and the non-differentially co-expressed neuronal module, mod2, which showed a substantially stronger enrichment of 39% of FMRP targets (*P*_hypergeometric_=7.38 × 10^−110^; Supplementary Data 12 and Fig. 2e).

Recently, Steinberg *et al*. organized the FMRP target genes into distinct temporally expressed subpopulations affected by different classes of genetic variation associated with ASD^36^. Based on this classification, we found that mod1 was enriched for FMRP targets expressed in the synapse during adolescence and adulthood (*P*_hypergeometric_=4.66 × 10^−4^), whereas mod2 was enriched for the FMRP targets in the modules that were expressed during fetal development (*P*_hypergeometric_=3.32 × 10^−4^; Supplementary Data 12 and Fig. 2f). Thus, we again find evidence that the genetic signal is stronger in the non-differentially expressed module (mod2), with a twofold enrichment for FMRP targets compared with the differentially expressed mod6, whereas no enrichment was observed for mod1. Incorporating the temporal data leads to a hypothesis that one important mechanism of action at the neuronal level is that primary mutations may occur in genes important in fetal development (captured by mod2), and altered expression of those genes would not be captured in the current study, where the youngest individual was 2 years of age. These mutations may lead to developmental changes reflected in adolescent and adult expressed genes showing differential expression between cases and controls (mod1 and mod6).

In this study, we provide transcriptomic evidence for type I interferon and M2-activation state abnormalities in autism that may lead to a variety of pathologic and phenotypic consequences. We further note that there is a strong negative correlation between two differentially co-expressed modules, mod5 (activated M2-state microglia genes) and mod1 (synaptic transmission genes; *r*=−0.92, Supplementary Data 14). Recently, microglia have been identified as cells capable of restoring neural function in the ASD-model *MECP2* knockout mice^37^. We observe, for the first time, that M2-activation state microglia genes, in particular, are altered in autism, potentially driven by type I interferon responses. This process may drive changes in neural progenitor cell proliferation and connectivity with resultant altered activity-dependent neural expression profiles in post-natal development^38^,^39^. The linkage of this pathway to autism may lead to more accurate and predictive models of idiopathic disease that might contribute to the identification of effective therapeutic approaches.

## Methods

### Brain tissue samples

*Brain tissue*. Frozen brain samples were acquired through the Autism Tissue Program (http://www.autismbrainnet.com), with samples originating from two different sites: the Harvard Brain Tissue Resource Center and the NICHD Brain and Tissue Bank at the University of Maryland. Tissue was obtained from post-mortem and written informed consent was obtained from next-of-kin or a legal guardian. This work was approved by the Institutional Review Board of the Johns Hopkins Hospital and University of Alabama at Birmingham and was conducted in accordance with institutional guidelines. The brain samples were dissected to obtain the cerebral cortex Brodmann area (BA) 19, anterior prefrontal cortex (BA10) and a part of the frontal cortex (BA44). Multiple cortical tissues corresponding to BA19, BA10 and BA44 were sequenced in 62, 14 and 28 samples, respectively, resulting in a total of 57 (40 unique individuals) control and 47 (32 unique individuals) autism samples. The average age at time of death of the 40 control and 32 autism individuals was similar (cases median age=20 years, controls median age=17 years), and there was no significant difference in cause of death between the two groups. Supplementary Data 1 contains the details of the corresponding subject phenotypes and additional characteristics.

### RNA library preparation and RNA sequencing

RNA-Seq libraries were prepared from 50 μg of total RNA from post-mortem brain tissue extracted with Trizol reagent according to the manufacturer’s protocol (Invitrogen). The TruSeq RNAseq kit (Illumina) was used with minor modifications as follows. Total RNA pools were subjected to two rounds of hybridization and elution with oligo(dT) dynabeads (Invitrogen) to obtain purified polyadenylated (polyA) RNA. After mRNA selection, samples were randomly fragmented to minimize bias at the 3′ end of the transcript. First-strand cDNA synthesis was performed using random primers (Illumina) and SuperScriptII Reverse-Transcriptase (Invitrogen) followed by second strand cDNA synthesis using RNaseH and DNA polymerase I (Illumina). Illumina supplied adaptors (TruSeq kit) were ligated to the purified, end-repaired and 3′ adenylated cDNA, and we performed manual 200 bp size-selection of the final product by gel-excision. The 200-bp cDNA template molecules were then amplified by PCR to create the final library. Quality control measures during library amplification included PCR from reactions with no template, from libraries made with no ligase (hence no adaptors) and finally from libraries with no adaptor oligonucleotides included in the ligase reaction. In these cases, the library failed to amplify, thereby ensuring specificity of the expected product for each run. Each library was evaluated for uniformity on a 2100 Bioanalyzer (Agilent) before sequencing on a single lane of Illumina’s HiSeq 2000 to produce 100 base pair (bp) single-end reads. Each sequencing run included samples randomized by sex, collection site and case–control status.

### Mapping and gene summarization of data from RNA-Seq

The sequenced reads for each sample were obtained as fastq files for 110 samples. To improve mapping, reads were trimmed to remove stretches of terminal A or Ts (*N*=3–12) and contaminating adaptor sequences using a Python script, `cutadapt' (v1.2.1)^40^. The sequenced reads were mapped using Tophat2 (refs 41, 42). Only uniquely mapped reads with a maximum of three mismatches were used to estimate gene counts. The RNA-Seq reads were mapped to a set of sequences derived from the Genome Reference Consortium Human build 37 (GRCh37) assembly, recommended by the 1000 Genomes Project^43^. Gene expression estimates were made for approximately 48,260 of the total 62,069 reported Ensembl gene annotations (GRCh37 or Human release 70), recommended by Kim *et al*.^42^, using the Python script ‘HTSeq-count’ (model type—intersection strict, http://www-huber.embl.de/users/anders/HTSeq/)^44^.

### Normalization of gene estimates

Subsequent to mapping, the gene count data were normalized for within and between lane biases (e.g., GC content) and sequencing depth by methods implemented in Conditional Quantile Normalization^45^ and Exploratory Data Analysis and Normalization for RNA-Seq (EDASeq)^46^, using the default settings for each method. We present the EDASeq-normalized data, and for a detailed discussion about the differences between EDASeq and Conditional Quantile Normalization, see Ellis *et al*.^16^.

We assessed summarized values on a per-gene basis, removing gene estimates for samples whose gene expression values were more than three standard deviations (s.d.) from the mean expression of each gene (per-gene outlier), as these outliers are artefactual in origin^16^.

### Quality assessment

Picard (http://picard.sourceforge.net, v1.87) command-line tools ‘CollectRnaSeqMetrics’ and ‘CollectGcBiasMetrics’ were used to provide RNA-Seq summary statistics (Supplementary Data 2). Six samples with low gene coverage (>20% of the 48,260 genes had zero coverage) were dropped from all downstream analyses, resulting in 104 samples. In addition, to detect global sample outliers because of technical or biological reasons, we used principal component analysis and identified a subset of 2,582 genes with at least ten reads per sample using the *`prcomp'* function in the `stats' package in R (http://www.R-project.org/). All 104 samples were within three s.d. of the mean of the first six principal components, which together explained ~55% of the variance^16^.

The RNA-Seq sequencing statistics on all 110 samples are detailed in Supplementary Data 2.

### Single-gene differential expression analysis

After normalization and outlier removal, ISVs^47^ were generated on a subset of 2,500 genes with at least ten read coverage in each sample. Data decomposition was performed on the log2 scale for the 2,500 genes. ISVs were generated while protecting for case–control status using the ‘isvaFn’ function in the ‘isva’ package in R. Differential gene analysis was performed using a subset of 13,262 genes that had at least three reads per sample across 90% of the samples.

A linear mixed regression framework was utilized to identify differential gene expression between 57 controls and 47 cases. To remove unwanted sources of variation while protecting differences because of the primary variable of interest (case–control status), site of sample collection, age, sex, brain region and ISVs were included as fixed effects. In addition, the model included a random intercept term to account for the correlation of gene expression estimated from multiple brain regions obtained from the same individual.

Permutation testing was used to estimate the threshold for transcriptome-wide significant differential expression (EDASeq, 400 permutations, *P*=4.76 × 10^−7^). We reiterate that the samples were obtained from two collection sites, and to estimate the threshold for transcriptome-wide significant differential expression, we permutated the case–control status within each site, maintaining the same phenotype for multiple samples (that is, brain regions) derived from a single individual.

We assessed the possibility of confounding in the expression of the two differentially expressed genes by investigating the expression stratified by sample collection site (Supplementary Fig. 1a,d). The sequencing coverage of *MAL* was calculated across the 4 exons and for 21 exons of *C11orf30* using ‘coverageBed’ from bedtools^48^ (Supplementary Fig. 1b,e). We also investigated for the expression of *MAL* and *C11orf30* during development and across different brain regions from Brainspan (http://hbatlas.org/pages/hbtd) (Supplementary Fig. 1c,f).

### Single-exon differential expression analysis

Exon-level estimates were obtained using the ‘count.py’ script from DEXSeq^49^ for each of the 104 samples. Exons with more than three reads across 90% of all samples were included for analysis. These 21,310 exons were modelled utilizing a linear mixed regression framework to identify differential exon usage between the 57 controls and the 47 cases. Site of sample collection, age, sex, brain region and ISVs (generated from the single gene-level analysis) were included as covariates to account for unknown confounding factors as fixed effects. In addition, the model included a random intercept term to account for the correlation of gene expression estimated from multiple brain regions obtained from the same individual.

### Single gene: GO and pathway enrichment analysis

To determine a common functional relationship among the top differentially expressed genes, we tested for the enrichment of biological processes using GO annotations (ftp://ftp.ncbi.nlm.nih.gov/gene/DATA/gene2go.gz). The number of genes differentially expressed at *P*<0.05, *P*<0.01, *P*<0.001 and *P*<0.0001 were 1,964, 749, 185 and 50, respectively. For each *P* value cutoff, we generated 2,000 random gene sets of equal size (that is, 1,964 for *P*<0.05) and performed the same enrichment analysis as on the original data set. Minimum *P* values for each enrichment analysis were stored. The 0.05 FWER was then calculated to estimate false positives by setting the 100th (out of 2,000) best *P* value as the threshold for a true discovery.

We also used an alternate method for pathway enrichment analysis for the identification of common functional categories represented by GO and curated gene sets. The pathways include all the pathways in GO and curated gene sets, which can be downloaded from MsigDB (http://www.broadinstitute.org/gsea/msigdb/collections.jsp#C1). The *P* value of each gene was determined from a linear mixed model. We then mapped these *P* values to non-negative z-scores(z_i), where




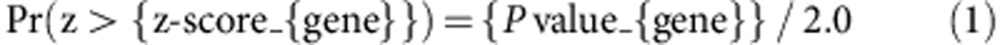




assuming the *P* values were two-tailed. Then for each pathway, we calculated the *P* value for a one-sided *t*-test of *z_i in the pathway ≥z_i not in the pathway*. We refer to this as the ‘pathway enrichment test’.

To account for false positives, we first generated 100 sets of balanced permutations, where for each permutation, the permuted case and control groups contain equal number of case and control samples from the original data set. Then, for each permuted data set, we did the same gene set enrichment test as we did with the original data and stored the *P* values for each pathway in each permuted data set. We then extract the best *P* value for each permutation, ranked them and set the 5th best *P* value as the threshold for a true discovery. This gives us a 0.05 FWER.

We also tested whether the genes associated with the risk of autism and ID were differentially expressed. Gene lists are in Supplementary Data 7, and details on the gene lists are provided below. The enrichment of the gene sets categories in the differentially expressed genes was tested based on the hypergeometric distribution model. Four lists of differentially expressed genes at *P* values <0.001,<0.005,<0.01 and<0.05, with 185, 494, 749 and 1,964 genes, respectively, were generated. The percentage of genes in each gene-set and *P* value corresponding to FWER=0.05 are tabulated in Supplementary Data 8a. We performed the ‘pathway enrichment test’ using equation 1 that does not rely upon defining a differentially expressed set of genes, broadly looking for differential expression (without a *P* value cut-off) among genetically associated genes (Supplementary Data 8b). We obtained the *P* values at FWER<0.05 (100 permutations) as described above.

### Artefact corrected data set

For the single-gene differential expression analysis, we used the ISVs to correct for both technical and biological confounders in the expression data. For the differential co-expression analysis, we identified various sequencing artefacts, computed by Picard command-line tools, which were largely confounding the expression data on the EDASeq-normalized genes (Supplementary Data 3a). The correlations between the various sequencing artefacts are provided in Supplementary Data 3b. We used a multivariate linear regression model to correct the gene expression estimates of sequencing artefacts (SAs), collection site (CS), sex (*S*), age (*A*) and brain region (BR), yielding an artefact corrected (AC) data set. The sequencing artefacts used in the model were ‘percent coding bases’, ‘percent utr bases’, ‘percent intronic bases’, ‘percent intergenic bases’, ‘median CV coverage’, ‘median 5′ to 3′ bias’, ‘aligned reads’ and ‘AT dropout’. The correction formula was (equation 2) as follows assuming we are correcting for only two sequencing artefacts:




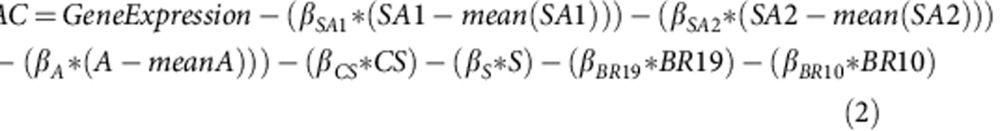




### Combined co-expression analysis

We investigated the entire AC data set to obtain gene sets or modules that were differentially co-expressed between autism cases and control brains using WGCNA^18^. We used WGCNA’s ‘signed’ co-expression measure to construct the interconnected gene modules to track the sign of the co-expression information^18^. Pearson’s correlations were calculated between 13,443 genes in the 104 samples. The WGCNA method transforms the correlation values to an adjacency matrix using a power function. This power function is selected based on a fit to scale-free topology, and a threshold of 9 (scale-free *R*^2^ of 0.7) was chosen in this study. This power function weights the network by transforming the pairwise correlation values and computing pairwise topological overlap (TO) between genes^18^. TO is a measure of connection strength between genes. Genes with high TO are clustered into co-expression modules. Each group of interconnected genes is co-expressed and the module is represented by the ME (the first principal component of the module). The connectivity of every gene in every module is represented by correlation to the ME, kME. In this study, this intramodule strength (kME) was ≥0.45 for all the modules.

Once the co-expression modules were created, they were numerically labelled by module size, with mod1 denoting the largest module. The co-expression analysis on 13,443 genes from the AC data identified 12 modules, with each module being represented by its first principal component or eigengene (for example, ME1) for each sample. We tested the association of each eigengene with case–control status using a univariate linear mixed regression model, with a random intercept term to account for the correlation among multiple samples derived from the same individual. No additional variables are included in the analysis, as the modules were constructed using the AC data (described above). The multiple test correction threshold using the Bonferroni method was 4.0 × 10^−3^. The permutation threshold, *P*<2.0 × 10^−3^, was determined by permuting case–control labels (*n*=100 permutations) for the eigengene values and re-running the regression analysis. The fifth lowest *P* value was deemed as the study-wide empirical threshold for *P*<0.05.

Most of the 13,443 genes were clustered into mutually exclusive co-expressing modules. However, 5,075 genes were assigned into the predefined mod0, which is reserved for non-module genes. All 13,443 genes and their module membership along with the correlation of the genes to each module (kME) are tabulated in Supplementary Data 10. The Pearson’s correlation between modules is shown in Supplementary Data 14, and the robustness of correlations was assessed using bootstrap with replacement analysis.

### Stratified co-expression analysis

We investigated the global similarity in transcriptome organization in the autism case and control brains by constructing signed networks for the autism case and control brains separately (power function threshold of 9). The construction of the signed networks separately for cases and controls identified 18 modules for each. We utilized the module preservation statistic Zsummary^50^, described in the ‘modulePreservation’ R function implemented in WGCNA, to assess the overlap in network modules obtained from the autism case and control brain data sets. The Zsummary statistic takes into account the overlap in module membership, the density (mean connectivity) and connectivity (sum of connections) patterns of modules. We adopted the recommended significance thresholds: Zsummary <2 implies no evidence for module preservation, 2> Zsummary <10 implies weak to moderate evidence, and Zsummary >10 implies strong evidence for module preservation. Using the recommended thresholds, we clearly observe that all 18 modules are conserved between the case and control brains (Zsummary >2, Supplementary Data 9).

### GO analysis

We functionally annotated the 12 modules with GO terms. The enrichment of the GO terms in each of the 12 modules was evaluated based on the hypergeometric test. To account for false positives, 12 random modules of the same size were generated 2,000 times, the hypergeometic test was carried out, and the 0.05 FWER was calculated. We have tabulated the GO term enrichment for each module at FWER=0.05 in Supplementary Data 13a–k.

### Gene list enrichment analysis

To help provide insight into the interpretation of the gene expression data, we compiled gene sets that have been either implicated in ASD^5^ or have been designated as markers for specific cell types^51^,^52^,^53^. The main lists in this study are provided in Supplementary Data 7, along with sources for each list (gene lists are available at http:// www.arkinglab.org/resources). We present the enrichment of each module, with significance calculated based on the hypergeometric model and implemented in the GeneMerge software package^54^. To account for false positives, the 0.05 FWER was calculated as described above. The enrichment analysis with each module’s *P* value corresponding to FWER=0.05 is tabulated in Supplementary Data 12.

### Compilation of genetic association genes

As previously discussed with the single-gene analysis, the genes associated with autism and ID were taken from numerous sources presented in Supplementary Data 7. The genetic association is presented as independent but not mutually exclusive lists: (i) 155 genes (ASD SFARI 2012) were compiled by Parikshak *et. al.* and are a manually curated set of candidate genes implicated by common variant association, candidate gene studies, genes within ASD-associated CNV, and, to a lesser extent, syndromic forms of ASD. This list from the SFARI AutDB was restricted to genes with strong genetic evidence by also filtering by the category S (syndromic) and evidence levels 1–4 (1=high confidence, 4=minimal evidence). The ASD SFARI 2012 list excludes any exome sequencing-implicated RDNV genes; (ii) 235 genes (ASD SFARI 2014) from the SFARI AutDB database^22^ (accessed on July 2014). The list was restricted to genes with strong genetic evidence by filtering by the category S (syndromic) and evidence levels 1–4 (1=high confidence, 4=minimal evidence); (iii) 197 genes (ASD SFARI 2014 CV) are a subset of the ASD SFARI 2014 after removing the 896 genes with rare *de novo* variant from the four whole-exome sequencing publications^9^,^10^,^11^,^12^; (iv) Pinto *et al*.^33^ compiled a list of 124 genes (ASD [Pinto]) that have been implicated in ASD and was updated from a list provided by Betancur in 2011 (ref. 55). All of the 124 genes have also been implicated in ID. Only autosomal (AD) or X-linked (XL) genes were included. The genes and loci were included only if there was independent evidence from other studies^46^; (v) 896 RDNVs associated with autism were compiled by Parikshak *et al*. from four whole-exome sequencing publications^5^,^7^,^8^,^23^. (vi) Steinberg *et al*.^36^ compiled a list of genes disrupted by *de novo* nonsense, frameshift or splice-site point mutations in autism probands that were obtained from Iossifov *et al*.^5^ (59 genes; referred to as ‘I-exomes’) and three other recent studies by Sanders *et al*.^8^, O’Roak *et al*.^7^ and Neale *et al*.^23^ (65 genes combined from all three; referred to as ‘SON-exomes’); (vii) A list of genes disrupted by breakpoints of balanced chromosomal abnormalities (BCAs) observed in individuals with ASD was obtained from Talkowski *et al*.^56^ (32 genes; referred to as ‘T-BCAs’).

### Other gene list compilations

Voineagu *et al*. identified two co-expression gene modules that were dysregulated in post-mortem ASD brains, **asdM12** (a neuronal module, enriched for ASD associated genes) and **asdM16** (enriched with astrocyte, activated microglial markers, with functional annotation immune response, but no enrichment for ASD associated genes)^10^.

FMRP and its interacting partners (**FMRP interacting**) implicated with translational regulation of synaptic proteins^35^ and shown to be enriched with diverse class of ASD variants^57^. In additionally, Steinberg *et al*.^36^ demonstrated that the 832 FMRP-interacting partners, particularly in two modules (represented as **FMRP 1** and **FMRP 2**), exhibit differential temporal expression: genes in FMRP 1 tend to be specifically upregulated during fetal development, whereas genes in FMRP 2 were generally upregulated in adolescence and adulthood. Numerous cell type markers were tested, as presented in Supplementary Data 7 and 12. Finally, Uddin *et al*. identified 3,955 exons mapping to 1,744 genes with high expression in the brain and a low burden of rare mutations, and designate these as ‘brain-critical exons’^25^.

## Additional information

**How to cite this article:** Gupta, S. *et al*. Transcriptome analysis reveals dysregulation of innate immune response genes and neuronal activity-dependent genes in autism. *Nat. Commun.* 5:5748 doi: 10.1038/ncomms6748 (2014).

**Accession codes:** The RNA-Seq data have been deposited in the National Database for Autism Research (NDAR) under the accession code NDARCOL0002034.

## Supplementary Material

Supplementary Figures and References Supplementary Figures 1-3 and Supplementary References

Supplementary Data 1 List of sample characteristics and phenotypes

Supplementary Data 2 Samples with sequencing statistics as obtained from Picard tools

Supplementary Data 3 3a: Correlation (r, spearman correlation) of the ISV with sequencing artifacts.3b: Correlation (r, spearman correlation) between the sequencing artifacts metrics.

Supplementary Data 4 Differentially expressed genes.

Supplementary Data 5 Differentially expressed exons.

Supplementary Data 6 6a: Enrichment analysis of 749 genes differentially expressed at P < 0.01.6b: Enrichment analysis of 1964 genes differentially expressed at P < 0.05. 6c: Enrichment analysis of 185 genes differentially expressed at P < 0.001. 6d: Enrichment analysis of 50 genes differentially expressed at P < 0.0001

Supplementary Data 7 Gene lists

Supplementary Data 8 8a: Enrichment analysis of 1964, 749, 494 , 185 genes differentially expressed at P < 0.05, P < 0.01, P < 0.005, P < 0.001 , respectively 8b: Gene Set enrichment analysis to evaluate expression at the level of genetically associated genes.

Supplementary Data 9 Module conservation between co-expressed modules constructed separately in cases and controls

Supplementary Data 10 Gene and kME in different modules

Supplementary Data 11 Association of co-expression modules with disease status

Supplementary Data 12 Modules and the enrichment of gene sets

Supplementary Data 13 13a. mod1 and enrichment of Gene Ontology categories with FWER < 0.05. 13b. mod2 and enrichment of Gene Ontology categories with FWER < 0.05. 13c. mod3 and enrichment of Gene Ontology categories with FWER < 0.05. 13d. mod4 and enrichment of Gene Ontology categories with FWER < 0.05. 13e. mod5 and enrichment of Gene Ontology categories with FWER < 0.05. 13f. mod6 and enrichment of Gene Ontology categories with FWER < 0.05. 13g. mod7 and enrichment of Gene Ontology categories with FWER < 0.05. 13h. mod8 and enrichment of Gene Ontology categories with FWER < 0.05. 13i. mod9 and enrichment of Gene Ontology categories with FWER < 0.05. 13j. mod10 and enrichment of Gene Ontology categories with FWER < 0.05. 13k. mod11 and enrichment of Gene Ontology categories with FWER < 0.05.

Supplementary Data 14 Correlation between the 12 module eigengenes (ME) in the network
