# Supplementary material for: Transcriptome analysis reveals dysregulation of innate immune response genes and neuronal activity-dependent genes in autism
Source: Nat Commun. 2014 Dec 10;5:5748. doi: 10.1038/ncomms6748 (PMC4270294; doi:10.1038/ncomms6748)
Supplement: Supplementary Figures and References — Supplementary Figures 1-3 and Supplementary References [file ncomms6748-s1.pdf]

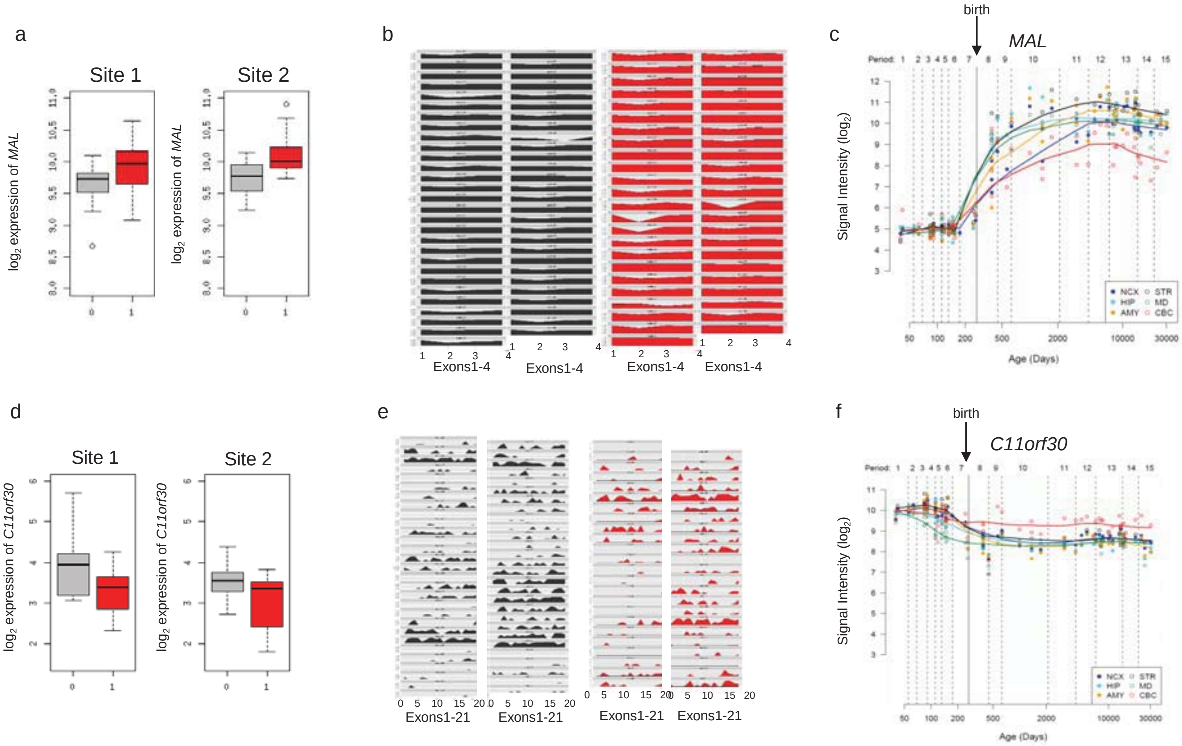


**Supplementary Figure 1** Transcriptome-wide top hits. (**a,d**) Expression levels of *MAL* (**a**) and *C11orf30* (**d**) in controls (grey) and cases (red) stratified by collection site demonstrate that the association with autism is consistent across collections sites. (**b,e**) A continuous plot with the coverage on a scale of 0-1 in all the 57 controls (grey) and 47 cases (red) across the 4 exons for *MAL* (**b**) and 21 exons for *C11orf 30* (**e**). (**c,f**) Expression of *MAL* (**c**) and *C11orf30* (**f**) through human brain development from post-conception week (PCW) 4 to 82 years (age on the x-axis and $\log_2$ expression of the gene across the different regions of the brain on the y-axis). The expression of *MAL* increases after birth in all brain regions, whereas *C11orf30* decreases after birth in all brain regions other than the amygdala (data from brainspan, http://www.brainspan.org).

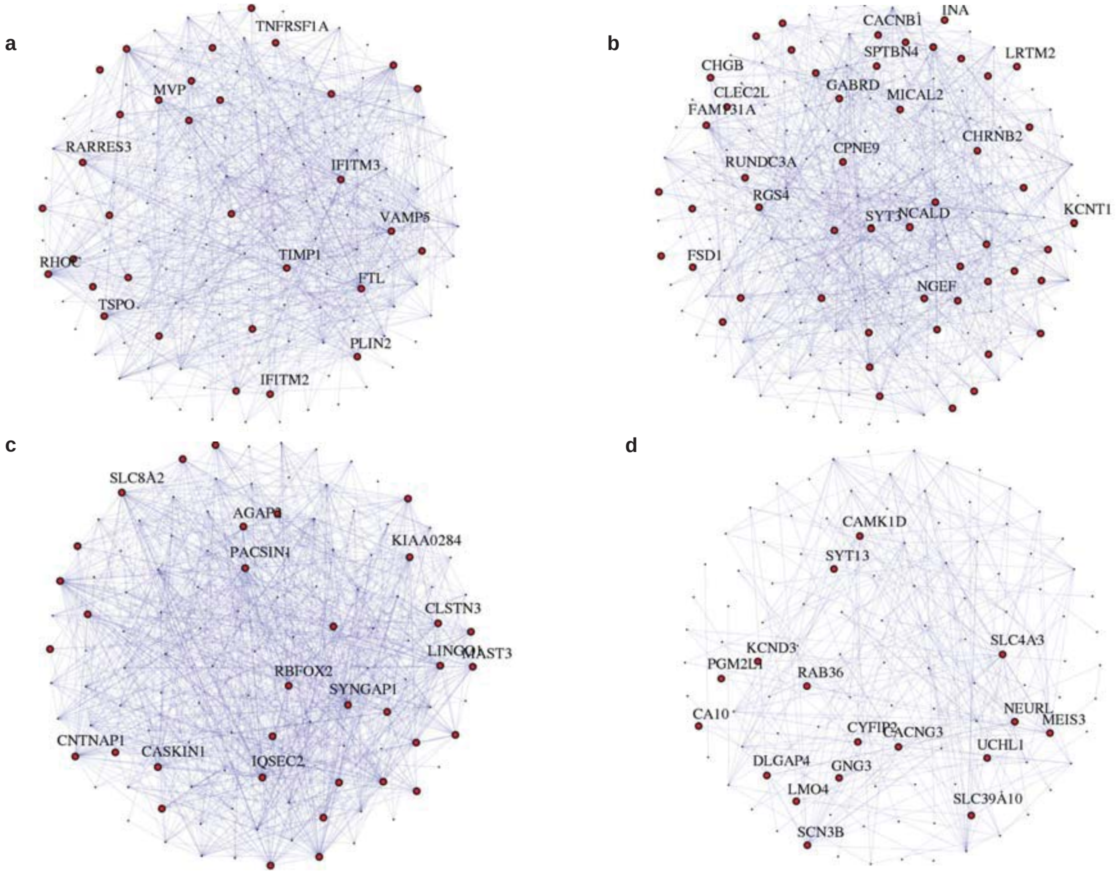


**Supplementary Figure 2** Visualization of the (**a**) mod5, (**b**) mod1, (**c**) mod2, and (**d**) mod6 modules. The top 150 connections for each module are represented as nodes. Genes with the highest correlation with the module eigengene value are represented by large node sizes.

**a**

| Category | % TOTAL (number of genes): | mod1 (91) | mod2 (68) | mod6 (30) |
|---|---|---|---|---|
| signaling proteins | | 7.7 | 17.6 | 36.7 |
| synaptic proteins | | 17.6 | 29.4 | 16.7 |
| ion channels | | 40.7 | 22.1 | 16.7 |
| calcium signaling | | 5.5 | 0.0 | 3.3 |
| glycine signaling | | 1.1 | 0.0 | 0.0 |
| GABA | | 9.9 | 5.9 | 3.3 |
| metabolism | | 2.2 | 1.5 | 0.0 |
| glutamate receptor | | 5.5 | 16.2 | 6.7 |
| hyperpolarization receptor signaling | | 2.2 | 0.0 | 0.0 |
| structural | | 2.2 | 2.9 | 3.3 |
| proliferation | | 1.1 | 0.0 | 0.0 |
| hormonal signaling | | 2.2 | 1.5 | 13.3 |
| cholinergic signaling | | 2.2 | 2.9 | 0.0 |

**b**

GeneGO Process- mod1 Synaptic Transmission

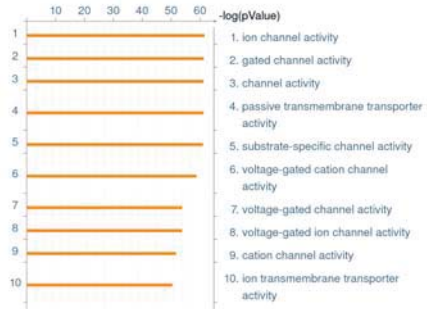


**c**

GeneGO Process- mod2 Synaptic Transmission

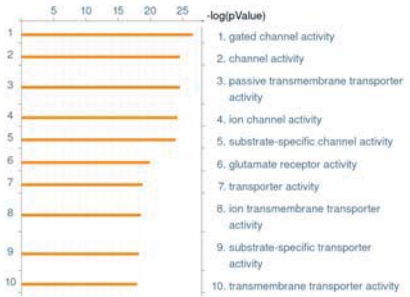


**d**

GeneGO Process- mod6 Synaptic Transmission

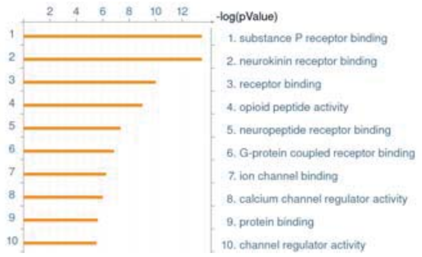


**Supplementary Figure 3 '**Synaptic Transmission' GO genes in mod1, mod2 and mod6. (**a**) Categorical assignments of genes within the 'Synaptic Transmission' GO term of mod1, mod2 and mod6. (**b**) Enrichment of ion channel activity (GABA-related) in mod1. (**c**) Enrichment of synaptic and glutamate receptor signaling in mod2. (**d**) Enrichment in peptide and hormone signaling in mod6. mod1 is down-regulated in autism, mod2 and mod6 are upregulated in autism (**Supplementary Data 11 and Fig. 2**).

**Supplemental References**


1. Cahoy, J. D. *et al.* A Transcriptome Database for Astrocytes, Neurons, and Oligodendrocytes: A New Resource for Understanding Brain Development and Function. *J. Neurosci.* **28,** 264–278 (2008).
2. Miller, J. A., Horvath, S. & Geschwind, D. H. Divergence of human and mouse brain transcriptome highlights Alzheimer disease pathways. *Proc. Natl. Acad. Sci. U.S.A.* **107,** 12698–12703 (2010).
3. Miller, J. A., Oldham, M. C. & Geschwind, D. H. A systems level analysis of transcriptional changes in Alzheimer's disease and normal aging. *J. Neurosci.* **28,** 1410–1420 (2008).
4. Nagata, T. *et al.* Profiling of genes associated with transcriptional responses in mouse hippocampus after transient forebrain ischemia using high-density oligonucleotide DNA array. *Brain Res. Mol. Brain Res.* **121,** 1–11 (2004).
5. Bayés, À. *et al.* Characterization of the proteome, diseases and evolution of the human postsynaptic density. *Nat Neurosci* **14,** 19–21 (2011).
6. Parikshak, N. N. *et al.* Integrative functional genomic analyses implicate specific molecular pathways and circuits in autism. *Cell* **155,** 1008–1021 (2013).
7. Abrahams, B. S. *et al.* SFARI Gene 2.0: a community-driven knowledgebase for the autism spectrum disorders (ASDs). *Mol Autism* **4,** 36 (2013).
8. Pinto, D. *et al.* Convergence of genes and cellular pathways dysregulated in autism spectrum disorders. *Am. J. Hum. Genet.* **94,** 677–694 (2014).
9. Betancur, C. Etiological heterogeneity in autism spectrum disorders: more than 100 genetic and genomic disorders and still counting. *Brain Res.* **1380,** 42–77 (2011).
10. Steinberg, J. & Webber, C. The roles of FMRP-regulated genes in autism spectrum disorder: single- and multiple-hit genetic etiologies. *Am. J. Hum. Genet.* **93,** 825–839 (2013).
11. Voineagu, I. *et al.* Transcriptomic analysis of autistic brain reveals convergent molecular pathology. *Nature* **474,** 380–384 (2011).
12. Darnell, J. C. *et al.* FMRP stalls ribosomal translocation on mRNAs linked to synaptic function and autism. *Cell* **146,** 247–261 (2011).
13. Kang, H. J. *et al.* Spatio-temporal transcriptome of the human brain. *Nature* **478,** 483–489 (2011).
14. Uddin, M. *et al.* Brain-expressed exons under purifying selection are enriched for de novo mutations in autism spectrum disorder. *Nat. Genet.* **46,** 742–747 (2014).
